# Pharmacokinetics of tebipenem pivoxil used in children suffering from shigellosis: a pilot study in Bangladesh

**DOI:** 10.1038/s41598-024-83549-3

**Published:** 2024-12-30

**Authors:** Sharika Nuzhat, Md Ridwan Islam, Syed Jayedul Bashar, Subhasish Das, Rukaeya Amin, Firdausi Qadri, Farhana Khanam, Dilruba Ahmed, Patricia B. Pavlinac, Cindy X. Zhang, Samuel L. M. Arnold, Amy Newlands, Mohammod Jobayer Chisti, Tahmeed Ahmed

**Affiliations:** 1 Clinical and Diagnostic Services, International Centre for Diarrhoeal Disease Research, Bangladesh (icddr,b), 68, Shaheed Tajuddin Ahmed Sarani, Mohakhali, Dhaka, Bangladesh; 2 Nutrition Research Division, International Centre for Diarrhoeal Disease Research, Bangladesh (icddr,b), Mohakhali, Dhaka, Bangladesh; 3 Infectious Diseases Division, International Centre for Diarrhoeal Disease Research, Bangladesh (icddr,b), Mohakhali, Dhaka, Bangladesh; 4 Office of the Executive Director, International Centre for Diarrhoeal Disease Research, Bangladesh (icddr,b), Mohakhali, Dhaka, Bangladesh; 5 Department of Global Health, University of Washington, Seattle, USA; 6 Department of Pharmaceutics, University of Washington, Seattle, USA; 7 GSK, London, UK

**Keywords:** Tebipenem, Pharmacokinetics, Dysentery, Shigellosis, Under-5 children, Medical research, Drug safety, Pharmaceutics, Gastrointestinal diseases, Bacterial infection

## Abstract

With increasing antibiotic resistance in gram-negative bacteria, including those causing *Shigellosis,* evidence of safety and pharmacokinetics data on new oral antibiotics is crucial. We aimed to investigate the safety and pharmacokinetic properties of an oral carbapenem, tebipenem pivoxil, along with it’s ability to produce desired results in childhood shigellosis. This randomized pilot clinical trial was conducted at Dhaka Hospital, icddr,b in 2022 between May and September. Thirty suspected shigellosis cases aged 24–59 months were randomized across two treatment groups equally: tebipenem pivoxil and azithromycin. Pharmacokinetics of tebipenem was assessed among fifteen children who received tebipenem pivoxil using Noncompartmental analysis (NCA). Clinical (absence of fever, abdominal pain/tenderness, diarrhoea, blood in stool, or death before Day-3) and microbiological (absence of *Shigella* on Day-7 culture) success after the antibiotic interventions were also evaluated. Sociodemographic and clinical characteristics were comparable between the randomization arms. Twelve children, each in the azithromycin arm and tebipenem arm, were positive for *Shigella* by culture on enrolment. C_max_ values of 5053.3, 2546.0, and 3759.2 ng/mL were observed for plasma tebipenem on Day-0, 1, and 2 respectively. Clinical success was observed among seven participants in each arm while two in the azithromycin arm and three in the tebipenem arm failed microbiologically. The tolerability and efficaciousness of tebipenem pivoxil appear to be comparable to azithromycin in treating childhood shigellosis in Bangladesh. We recommend a larger clinical trial to determine non-inferiority of tebipenem in regards to the current treatment guidelines.

## Introduction

Diarrhoea is the 2nd leading cause of death among children under-5 and accounts for an estimated 370,000 global deaths^1^. The Global Burden of Disease Study estimated that approximately 90% of all diarrhoeal deaths occurred in South Asia and Sub-Saharan Africa, the regions that are highly prevalent in diarrhoeal diseases^2^. Around 7% of under-5 deaths are accountable to diarrhoeal diseases in Bangladesh^1,3^.

*Shigella spp* is the leading bacterial cause of diarrhoea and dysentery, responsible for approximately 163 million diarrhoea cases^4^. Approximately 94,000 children and 148,000 of all ages died in 2019 from *Shigella* in low and middle-income countries according to the estimate of the Institute for Health Metrices and Evaluation (IHME) Global Burden of Disease Study^5^. Nearly 91 million shigellosis cases were reported in Asia resulting in 414,000 deaths each year^6^. Previous studies conducted in Pakistan, Thailand, Bangladesh, China, Indonesia, and Vietnam have shown that the burden of shigellosis is higher among children aged < 5 years^7^. High fever, abdominal cramps, and frequent passage of stool containing mucus and blood are primary symptoms of shigellosis^8^. The infection causes ulceration, inflammation, and loss of intestinal barrier function. *Shigella flexneri* is the most dominant among these strains in the developing countries including Bangladesh (54%-60%)^9^. Concurrently, there has been a notable increase in the prevalence of *Shigella sonnei*, rising from 7.2% in 2001 to 25% in 2011 in Bangladesh^10^. *Shigella spp*. often cause self-limiting conditions that can also be managed with oral rehydration and antibiotics^11^. Children < 5 years are particularly vulnerable because of their poor personal hygiene, partially developed immune system, and lack of previous exposures^12,13^.

The World Health Organization (WHO) recommends oral ciprofloxacin or azithromycin as first-line and intravenous (IV) or intramuscular (IM) ceftriaxone as second-line therapy for children with shigellosis^14,15^. However, irrational antibiotic use in diarrhoea, and emerging antimicrobial resistance (AMR) to ciprofloxacin have compelled researchers and clinicians to use azithromycin as alternative first and/or second-line antibiotic^16–18^. Recently, a published article on antimicrobial susceptibility for childhood shigellosis evaluated gradual increase in resistance to antibiotics used for shigellosis^17^. The ongoing diarrhoeal disease surveillance system (DDSS) in Bangladesh (icddr,b) regularly monitors the susceptibility pattern of *Shigella* isolates. In response to increased resistance to ciprofloxacin, azithromycin has been adopted as the 1^st^ line treatment for shigellosis in children^17^.

The increasing trend of AMR against the current antibiotics led researchers to look for an alternative medication for treatment of shigellosis. Tebipenem pivoxil, an oral carbapenem and a prodrug that is converted to its therapeutically active form- tebipenem, could be used as an alternative treatment regimen as its safety is well documented for children^19–21^. Currently licensed for use in Japan, tebipenem pivoxil is better tolerated than infusions, and it has exhibited broad-spectrum action both in vitro and in vivo against a number of crucial Gram-positive and Gram-negative pathogens, including ESBL producing Enterobacteriaceae^22^. Rigorous pre-clinical studies have established tebipenem pivoxil as an efficacious treatment option against *Shigella* infection^23,24^. There is no clinical evidence on the use of tebipenem pivoxil in shigellosis.

In this study, we have evaluated the effect of tebipenem pivoxil on shigellosis as an alternative treatment regimen for childhood shigellosis and compared it with ongoing treatment regimen (azithromycin). We also evaluated the pharmacokinetics of tebipenem in children treated with tebipenem pivoxil (Supplementary Fig. 1).

## Results

Out of 2249 screened cases, we enrolled 30 children, 15 of whom were randomized to the tebipenem arm; 15 to the azithromycin arm. Sociodemographic characteristics, nutritional status, clinical symptoms, and duration of hospitalization in both the groups are illustrated in (Table 1).

**Table 1:** Socio-demographic and clinical characteristics of enrolled cases.

Variables		Azithromycin (n = 15)	Tebipenem (n = 15)
Age, months; (mean ± SD)		37 ± 8.04	39.2 ± 8.74
Sex, male; n (%)		9 (60)	10 (66.67)
Gestational age, weeks; (mean ± SD)		37.93 ± 1.71	38.53 ± 1.36
Mode of delivery, caesarean section ; n (%)		10 (66.67)	8 (53.33)
Father’s age, years; (mean ± SD)		34.8 ± 4.6	32.3 ± 1.04
Mother’s age, years; (mean ± SD)		27.1 ± 5.4	26.7 ± 3.35
Father’s education, n (%)	1–5 years	3 (20)	4 (26.67)
	6–10 years	4 (26.67)	3 (20)
	10–12 years	3 (20)	5 (33.33)
	> 12 years	5 (33.33)	3 (20)
Mother’s education, n (%)	1–5 years	1 (6.67)	3 (20)
	6–10 years	7 (46.67)	7 (46.67)
	10–12 years	6 (40)	1 (6.67)
	> 12 years	1 (6.67)	4 (26.67)
Exclusive breastfeeding for 6 months, yes; n (%)		14 (93.33)	14 (93.33)
Household monthly income, taka; median (IQR)		15,000 (14,000–31,000)	16,000 (12,000–20,000)
Locality where caregiver lives, urban; n (%)		10 (66.67)	13 (86.67)
Number of siblings, n (%)	0	5 (33.33)	8 (53.33)
	1	6 (40)	4 (26.67)
	2	4 (26.67)	2 (13.33)
	3	0 (0)	1 (6.67)
Birth order of the diseased child, n (%)	1st	6 (40)	7 (46.67)
	2nd	6 (40)	6 (40)
	3rd	3 (20)	1 (6.67)
	4th	0 (0)	1 (6.67)
Immunization status, complete, n (%)		15 (100)	14 (93.33)
First consulted with whom, n (%)	Registered physician	6 (40)	8 (53.33)
	Quack	2 (13.33)	0 (0)
	Local drug seller	6 (40)	6 (40)
	Others	0 (0)	1 (6.67)
	None	1 (6.67)	0 (0)
Use of antibiotics before admission to the hospital, n (%)	Yes	8 (53.33)	8 (53.33)
	No	7 (46.67)	6 (40)
	Didn’t know	0 (0)	1 (6.67)
Cough, yes; n (%)		2 (13.33)	0 (0)
Fever, yes; n (%)		8 (53.33)	12 (80)
Vomiting, yes; n (%)		4 (26.67)	1 (6.67)
Mid-upper arm circumference during enrolment, cm; (mean ± SD)		14.78 ± 1.14	14.78 ± 0.85
W/A Z score during enrolment, (mean ± SD)		− 1.46 ± 1.03	− 1.63 ± 0.58
W/H Z score during enrolment, (mean ± SD)		− 1.52 ± 0.95	− 1.66 ± 0.70
H/A Z score during enrolment, (mean ± SD)		− 0.87 ± 0.87	− 0.79 ± 0.93
Radial pulse during enrolment, (mean ± SD)		115.8 ± 13.70	110.07 ± 15.10
Respiratory rate during enrolment, (mean ± SD)		26.27 ± 3.99	25.93 ± 4.15
Temperature during enrolment, (mean ± SD)		37.02 ± 0.92	37.05 ± 1.20
Dehydration during enrolment, n (%)	No sign of dehydration	11 (73.33)	11 (73.33)
	Some dehydration	4 (26.67)	4 (26.67)

Nearly all (29/30) cases (14 in tebipenem pivoxil arm and 15 in azithromycin arm) completed the study.

### Culture-confirmed shigellosis cases

**Fig. 1:**
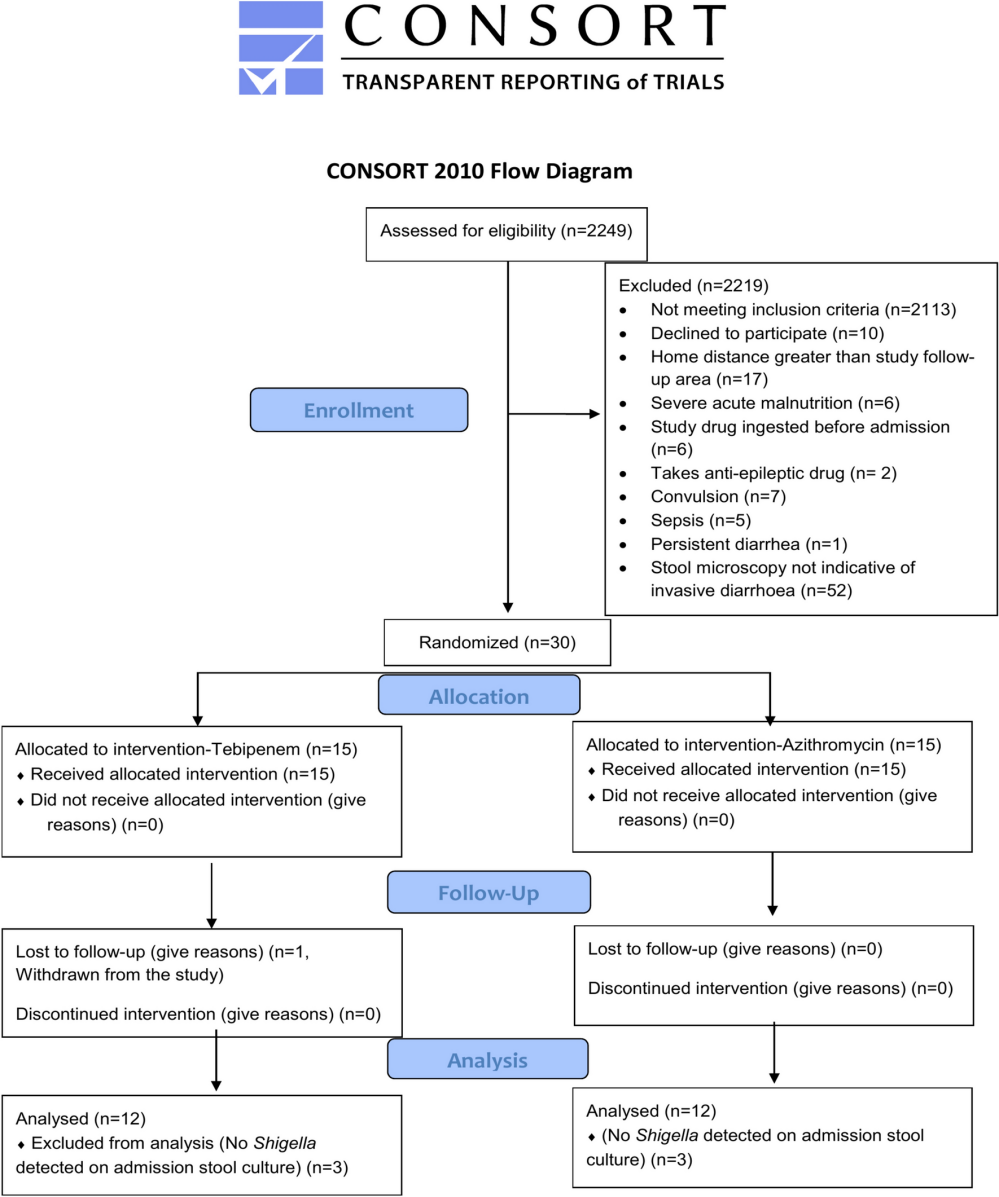
Consort Flow Diagram of the study**.**

24/30 cases had *Shigella* isolated from pre-randomization stool in microbiologic culture (12 in the tebipenem arm and 12 in the azithromycin arm) (Fig. 1). Among all shigellosis cases, 16/24 cases (66.7%) were culture-confirmed *Shigella flexneri*. On Day-7, 5/29 cases were positive with *Shigella* isolates where 60% (3/5) isolates were *Shigella sonnei* (Table 2)*.*

**Table 2:** Stool Culture results of enrolled children.

	Stool culture	Azithromycin	Tebipenem	Total
On admission	No *Shigella*	3	3	6
	*Shigella flexneri*	9	7	16
	*Shigella boydii*	0	1	1
	*Shigella sonnei*	3	4	7
				30
On Day 7	No *Shigella*	13	11	24
	*Shigella flexneri*	1	0*	1
	*Shigella boydii*	0	1	1
	*Shigella sonnei*	1	2	3
				29*

*one lost to follow up who was positive for *S. flexneri* on admission and was in tebipenem arm.

### Clinical and microbiological success with study interventions

Total 14 children, 7 in each arm were considered to have achieved clinical success. Microbiological success was better in azithromycin arm compared to tebipenem pivoxil group (10 cases vs 08 cases respectively).

### Clinical failures

There was a total of 10 children among culture positive *Shigella* cases (n = 24) who were labeled as clinical failures as all of them had visible blood in stool after completion of 48 h of antibiotic treatment. Both tebipenem and azithromycin arms had equal number (n = 5) of clinical failure cases.

### Microbiological failure

While analyzing for microbiological failure cases, we considered 11 in the tebipenem arm and 12 for the azithromycin arm. One child withdrew from the tebipenem arm and we did not have his stool on Day-7 to perform culture. We had total 5 microbiologic failure cases in this study, two in the Azithromycin arm and three in the Tebipenem arm**.**

### Changes in biochemical parameters

We performed Serum AST, ALT, creatinine, and total carnitine investigations after completion of antibiotic treatment. Total carnitine levels were slightly higher in tebipenem arm whereas other parameters seemed comparable and within normal range (Table 3).

**Table 3:** Biochemical parameters (post treatment) of enrolled cases in each arm.

	Azithromycin (n = 15)	Tebipenem (n = 15)
Day 3 AST, U/L; (median, IQR)	38.63 (32.14, 57.32)	38.94 (34.3, 47.09)
Day 3 ALT, U/L; (median, IQR)	14.47 (11.24, 30.68)	12.63 (08.68, 21.44)
Day 3 serum creatinine, µmol/L; (median, IQR)	25.35 (21.84, 26.93)	24.77 (23.18, 30.35)
Day 3 Total carnitine, ng/ml; (median, IQR)	118.35 (25.7, 211.7)	174.38 (111.13, 280.3)

*one lost to follow up who was positive for *S. flexneri* on admission and was in tebipenem arm.

### Safety and adverse events

We used the ‘*Division of AIDS (DAIDS) table for grading the severity of adult and pediatric adverse events, 2017’* to define the adverse events (AE) in our study^25^. We did not observe any significant AEs, for example, vomiting, rash, respiratory distress, or gastrointestinal symptoms, in our study, which may be related to the intervention. Similarly there were no hospitalizations during follow-up or deaths in either arm in our trial.

### Pharmacokinetic analysis

We assessed the pharmacokinetics of tebipenem in children who received tebipenem pivoxil. Each child was administered a 4 mg/kg/dose of tebipenem pivoxil three times a day for three days. The total daily dose (12 mg/kg/day) is the highest dose used in the clinic for the study population age range. Blood samples were collected at multiple time points over the three day period to determine tebipenem concentrations (Table 4).

**Table 4:** Blood collection timepoints for pharmacokinetic analysis.

Timepoints	Definition of timepoints
Day 0 (Enrolment day 1)	Before 1st Dose of Tebipenem (Pre-Dose 1)
Day 0 (Enrolment day 1)	After 30 min of 1st Dose of Tebipenem
Day 0 (Enrolment day 1)	After 1 h of 1st Dose of Tebipenem
Day 0 (Enrolment day 1)	After 4 h of 1st Dose of Tebipenem
Day 0 (Enrolment day 1)	After 1–2 h of 2nd Dose of Tebipenem
Day 0 (Enrolment day 1)	After 1–2 h of 3rd Dose of Tebipenem
Day 1 (Enrolment day 2)	Before 1st Dose of Tebipenem (Pre-Dose 1)
Day 1 (Enrolment day 2)	After 1–2 h of 2nd Dose of Tebipenem
Day 1 (Enrolment day 2)	After 1–2 h of 3rd Dose of Tebipenem
Day 2 (Enrolment day 3)	Before 1st Dose of Tebipenem (Pre-Dose 1)
Day 2 (Enrolment day 3)	After 30 min of 1st Dose of Tebipenem
Day 2 (Enrolment day 3)	After 1 h of 1st Dose of Tebipenem
Day 2 (Enrolment day 3)	After 4 h of 1st Dose of Tebipenem
Day 2 (Enrolment day 3)	After 1–2 h of 2nd Dose of Tebipenem
Day 2 (Enrolment day 3)	After 1–2 h of 3rd Dose of Tebipenem

**Fig. 2:**
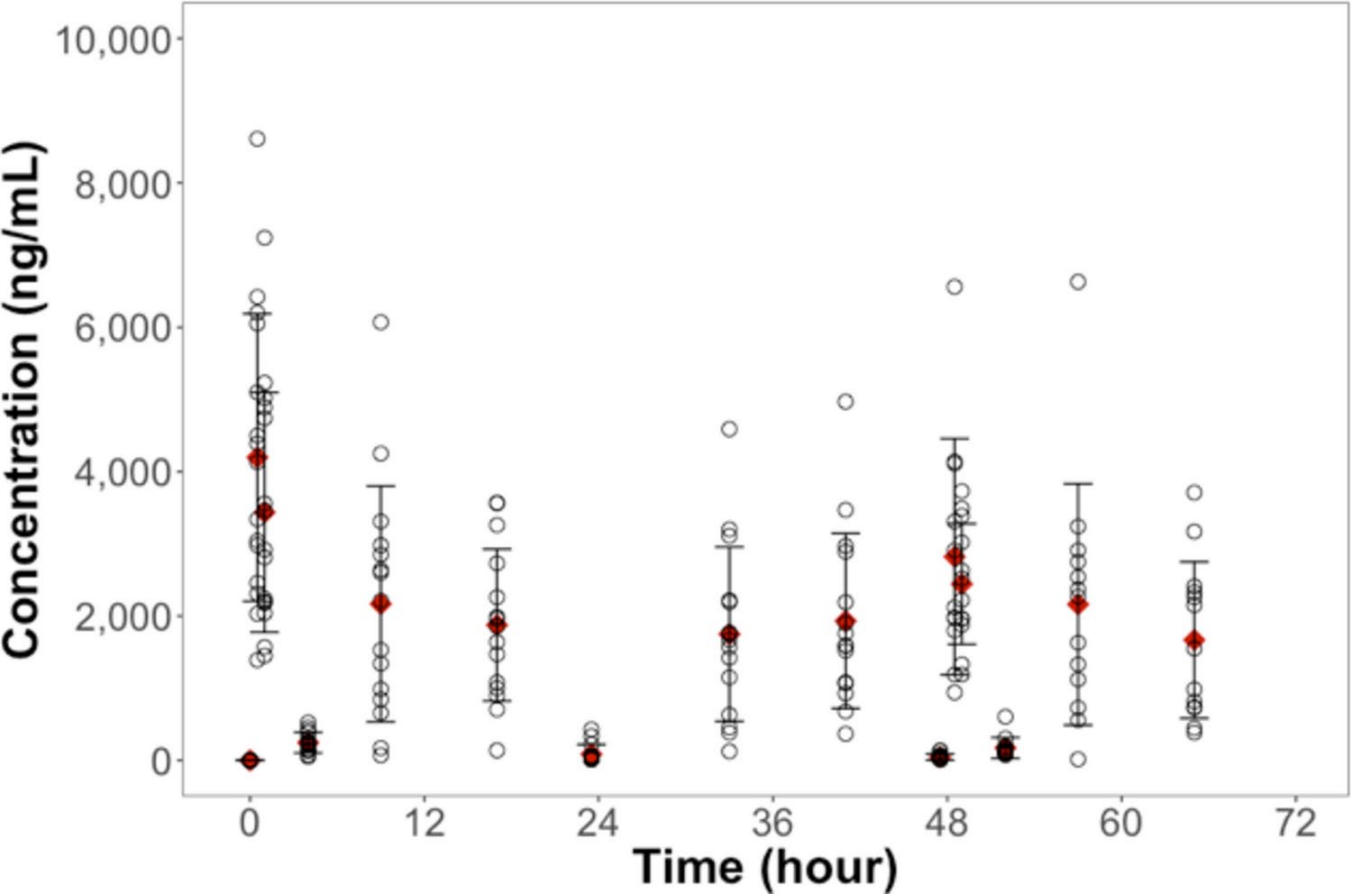
Tebipenem Plasma Concentration over Time.

Out of 225 targeted pharmacokinetic samples, we were not able to collect blood for 14 time points among four participants. The plasma concentration of tebipenem peaked within 1st hour following each dose for most study participants and then declined rapidly before the next dose (Fig. 2). C_min_ values are not reported because the exclusion of BQL values would skew the estimation for Days 0 and 1. Moreover, no pre-dose samples were collected on Day-2 to inform meaningful C_min_ estimation.

Open circles represent observed individual tebipenem concentrations. Red diamonds mark the mean concentration at each time point. Error bars represent one standard deviation around the mean.

NCA was performed to characterize important PK parameters for tebipenem after tebipenem pivoxil administration. C_max_ values of 5053.3, 2546.0, and 3759.2 ng/mL were observed for plasma tebipenem on Day 0, 1, and 2 respectively. Tebipenem systemic exposure (i.e., area under the curve (AUC)) over the four hours after the first dose on Day 0 (AUC_0-4_) and Day 2 (AUC_48-52_) were 8481.1 ng·hr/mL and 6101.8 ng·hr/mL, respectively (Table 5). The observed C_max_ values were similar to a mean C_max_ of 3.19 µg/mL and 3.63 µg/mL previously reported following 4 mg/kg twice daily dosing of tebipenem pivoxil in pediatric patients aged 0.5 to 3 years and 3 to 6 years, respectively^26^. No accumulation of tebipenem over the study period was observed, which agrees with the previously reported terminal half-life of approximately 1 h for tebipenem in pediatric patients^27^.

**Table 5:** Tebipenem pharmacokinetic parameter summary.

	Parameter	unit	N	Mean	SD	%CV	Geometric mean
Day 0	${C}_{max}$	ng/mL	15	5053.3	1590.2	31.5	4828.5
	${T}_{max}$	Hr	15	2.9	5.0	171.4	1.1
	${AUC}_{0-4}$	ng*hr/mL	15	8481.1	3378.0	39.8	7878.1
Day 1	${C}_{max}$	ng/mL	15	2546.0	1123.2	44.1	2339.0
	${T}_{max}$	Hr	15	38.4	3.9	10.1	38.2
Day 2	${C}_{max}$	ng/mL	13†	3759.2	1415.6	37.7	3542.4
	${T}_{max}$	Hr	13†	53.2	6.4	12.0	52.8
	${AUC}_{48-52}$	ng*hr/mL	13†	6101.8	2171.8	35.6	5712.4

† Two subjects were excluded from the analysis due to treatment termination on Day-2 (only the first 6 doses were administered).

## Discussion

In this pilot randomized clinical trial, we demonstrated feasibility and safety of tebipenem pivoxil as an oral antibiotic for shigellosis in children. We found that the number of clinical success cases in the tebipenem arm was equal to the numbers in azithromycin arm. Our trial demonstrated no adverse effect of tebipenem pivoxil among children with shigellosis.

Recently published systematic review and meta-analysis carried out on the burden of shigellosis in Southeast Asia revealed that under-5 children have considerable infections with *Shigella* spp. which is around 10% among *Shigella* positive cases of all age groups^28^. This high prevalence demands the need for interventional efforts focusing exclusively on children. Current WHO guidelines suggest the utilization of fluoroquinolones as the first-line treatment, followed by β-lactams and cephalosporins as second-line options for childhood shigellosis^29^. These recommendations align with current evidence and are consistent with other international guidelines. However, due to high resistance to fluoroquinolones (ciprofloxacin)^30^, some guidelines suggested the use of azithromycin as first-line therapy for shigellosis in children^17,31^. Shigellosis commonly spreads through person-to-person transmission^32^. Epidemics can be seen in areas with poor sanitation and overcrowding, and they have been extensively observed in environments like daycare centers, nursing homes, and custodial institutions for prisoners and individuals with mental disabilities. Controlling these outbreaks poses significant challenges^33,34^. Additionally, a gradual increase in resistance of antimicrobials against shigellosis is alarming worldwide^17,35–37^. Given that carbapenems serve as the last-resort treatment for numerous infections, underscoring the importance of mitigating the emergence of resistance. Tebipenem, widely known as Orapenem in Japan, is already licensed for treating pediatric respiratory infections and has established safety documentation^27,38^, and its oral administration capability^39^, makes it particularly well-suited for treating gastrointestinal infections within community settings.

Tebipenem demonstrates an outstanding in vitro profile against multidrug-resistant (MDR) and extensively drug-resistant (XDR) *Shigella* strains, as well as other Gram-negative bacteria^40^. In animal models of shigellosis, when animals were infected with ciprofloxacin-susceptible *Shigella* isolates, tebipenem exhibited higher success thus demonstrating comparable activity to ciprofloxacin^41^.

In this pilot trial, we demonstrated equivalent clinical success of tebipenem pivoxil and azithromycin for treatment of shigellosis in children. No children in the study developed any side effects like vomiting, diarrhoea, skin rash, urticaria, etc. due to the intervention. Total carnitine was slightly higher on Day 3 among the children who received tebipenem intervention in our study. β-lactam antibiotics like tebipenem can increase total carnitine concentrations due to pivalic moiety which reduces the carnitine level in skeletal muscle^42^. A study found that the differences in carnitine levels in urine and plasma, after intervention, were comparable to those of other antibiotics with the pivalic moiety, which have already been used clinically^43^. The study observed no muscle pain or weakness in any of the subjects which was similar to our findings^43^. Smaller sample size in our study had limited power to detect an impact on long term outcomes like linear growth.

Tebipenem pivoxil was found to be well tolerated among the children. Tebipenem pivoxil demonstrated desired results in this study with the dosage regimen of 4 mg/kg administered 3 times daily for 3 days in 7 children out of 12 (58%) who were culture positive for *Shigella* in pre-randomization stool. A recently published article reported that tebipenem effectively reduced the *Shigella* infection burden in mice infected with *Shigella* and gnotobiotic piglets, and the drug displayed ideal pharmacokinetic properties in the piglet model^41^. In our pharmacokinetic data, the drug level exposure in children also remained within the safety level limits.

Carbapenem resistance has not been reported in any *Shigella* species to date and is not currently prevalent among other Gram-negative bacteria that cause diarrhoea. Considering *Shigella*’s tendency to develop resistance to frequently used antimicrobials via the acquisition of antimicrobial resistance (AMR) plasmids and individual gene cassettes, it is important to also assess the necessity of a companion diagnostic. This evaluation is crucial to prevent treatment failures or excessive use of the drug, which could potentially stimulate resistance.

The results of this study should be interpreted considering the relatively small sample size and the characteristics of the study population, which predominantly consisted of shigellosis cases with the majority of whom were aged between 2 and 5 years old. The degree to which the results can be generalized to the larger community including elderly individuals, is not known. The trial is underpowered to detect important differences across intervention arms.

Several clinical trials undertaken in Kenya, Israel, and the USA reported that prophylactic treatment with bioavailable antibiotics effectively prevented shigellosis among individuals exposed to *Shigella* species^44–46^. Prophylactic antibiotic therapy is not recommended for the treatment of diarrhoeal syndromes, including shigellosis, due to concerns regarding adverse effects, drug interactions, and the potential for fostering bacterial resistance^47^. Thus, prophylactic use of tebipenem pivoxil in the prevention of shigellosis was also not evaluated for this population.

Due to the small number of *Shigella-*infected patients in the study, a larger trial is necessary to draw conclusive findings regarding the efficacy of tebipenem pivoxil in the treatment of drug resistant *Shigella* infection. Blood samples for PK analysis were not collected 8 h after dosing as the children were very sick and large number of blood draws in rapid succession was not possible. But we consider this as a limitation to the study.

In conclusion, the overall result of this pilot study establishes preliminary evidence and support for future evaluations of oral tebipenem pivoxil against *Shigella* infection in children. We observed a similar number of clinically successful cases in both arms. Given its oral bioavailability and safety profile, orally administered tebipenem pivoxil could be an ideal repurposing candidate if a larger trial provides the same result.

## Methods

### Study site

Between May 2022 and September 2022, the study was conducted at the Dhaka Hospital of icddr,b, renowned as the world’s largest treatment facility for diarrhoeal diseases. Annually, this hospital offers free treatment to roughly 150,000 patients, with approximately 60% of them are under-5 children.

### Study participants

Children between the ages of 24 to 59 months having clinical symptoms indicative of *Shigella* infection, such as fever, presence of mucus and/or blood in stools, tenesmus (straining during bowel movements), and stool microscopy revealing red blood cells (RBC) and leukocytes more than 10 per high power field (hpf), were identified upon initial presentation as eligible candidates for participation in the study. We collected written informed consent from parents who were interested in participating in the trial.

Children who had already received the study antibiotics (azithromycin, ceftriaxone, and/or tebipenem) for their current illness, as confirmed by bottle or prescription records, were excluded from this study. Additionally, children with severe acute malnutrition (SAM) or displaying signs of other infections requiring antibiotic treatment were excluded. Those for whom determining the objectives of the study drug was difficult were also excluded. Furthermore, patients with convulsive disorders, metabolic disorders, severe hepatic or renal dysfunction, or a history of β-lactam antibiotic allergy were not considered eligible for the study.

### Study procedures

Following informed consent, the randomized, open-labeled study comprised of a screening process during admission to the Dhaka hospital, icddr,b that occurred before randomization, administration of study drug in the in-patient ward after randomization, and follow-up visits on Day, 7 and 30 of post-randomization.

Following admission, children were screened for eligibility, and those suspected of having shigellosis were randomized to receive the local antibiotic azithromycin (10mg/kg per day) or oral tebipenem pivoxil (4mg/kg × 3 times x daily). Simple random sampling was employed for this pilot study where 15 children were allocated in each treatment arm. Children remained in the hospital ward for a minimum of 4 days after the start of treatment. Children were eligible to be discharged from the hospital after completion of 3 days of antibiotic treatment if there was no fever/abdominal pain/abdominal tenderness/diarrhoea/blood in stool.

Children who failed to show adequate response to the treatment within 48 h or worsening the symptoms after 24 h were switched to IV ceftriaxone (50 mg/kg/day) for another 3 days.

Stool samples were collected prior to randomization (Day-0), after completion of 3 days of treatment with azithromycin or tebipenem pivoxil (Day-3), on Day-7 and Day-30 of post-randomization.

The total study duration was 31 days, from Day-0 defined as enrolment day to Day-30 which was the last follow-up day/study completion day. All through the study duration, continuous monitoring was conducted among study children for any adverse events, defined as any unfavorable medical occurrences, irrespective of their suspected cause. Vital signs were assessed at minimum intervals of every 8 h during the inpatient phase. Clinical laboratory tests including aspartate transaminase (AST), alanine transaminase (ALT), serum creatinine, and serum carnitine levels were performed after completion of intervention on Day-3.

### Interventions

#### Tebipenem pivoxil

Tebipenem pivoxil was provided with 4 mg/kg/dose, thrice daily for 3 days. Tebipenem pivoxil from Meiji Seika Pharma Co., Ltd of Japan was used for this study.

The pediatric formulation of Tebipenem pivoxil, often known as Orapenem (10% fine granules), was administered after dissolving in distilled water.

#### Azithromycin

Azithromycin^48^: Azithromycin was provided with 10 mg/kg/dose, once daily for 3 days. The medicine was collected from a local pharmaceutical company and suspension of azithromycin was prepared with distilled water.

### Pharmacokinetics sampling and analysis

Blood samples for pharmacokinetics (PK) analysis were collected from the 15 children enrolled in the Tebipenem arm. A volume of 0.5 ml of blood was collected at each timepoint from each participant. Blood samples were centrifuged at 1006 × g immediately following collection, and 0.25 ml plasma was stored at − 80 ℃.

Tebipenem plasma concentrations were measured by Q2 Solutions (Ithaca, NY, USA) using liquid chromatography-tandem mass spectrometry (LC/MS/MS). Available PK concentrations were tabulated and graphed. Any PK sample concentrations flagged as laboratory errors were mentioned in the report and omitted from the analyses. Concentrations falling below the limit of quantification (LLOQ) at 30 ng/ml were referred to as below quantification limit (BQL).

### Outcome measures

The primary outcome of this pilot trial was clinical success, defined as the absence of all of the following clinical signs on Day-3 of follow-up (after completing 3 days of treatment): fever (which is defined by axillary temperature ≥ 38℃), abdominal pain/or tenderness (defined either pain localized by the child in response to inquiry from the parent/caregiver or the presence of any facial expression indicating discomfort during palpation of any part of the abdomen), diarrhoea (≥ 3 abnormally loose or watery stools passed in the last 24 h), blood in stool, or death prior to Day-3.

Secondary outcome for this was microbiologic success at Day-7, which was defined as the absence of Shigella *spp* isolated from microbiologic culture and *Shigella* DNA quantities (using Ct values from qPCR) equal to or higher than enrolment values (lower Ct values).

### Laboratory investigations

A portion of the stool sample from Day-0 was used for microscopy including faecal leukocyte determination. Stool samples were analyzed at the Clinical Microbiology Laboratory in icddr,b. For the isolation of *Shigella spp.* and *E. coli*, the stool samples were cultured on to Salmonella- Shigella (SS) agar and MacConkey (MAC) agar media respectively. Following overnight incubation at 35 ± 2 °C, the MAC and SS agar plates were examined for lactose-fermenting and non-lactose-fermenting colonies. Colonies suspected to be *Shigella spp*. and *E. coli* were inoculated into Kligler iron agar and motility indole urease agar media for further testing. After incubation for 18–24 h at 35 ± 2℃, the test media were examined for the characteristics of *Shigella spp.* and *E. coli* biochemical reactions. Slide agglutination for *Shigella* serotyping was further performed using commercially available *Shigella* antisera (Mast Group Ltd., UK) for the suspected *Shigella* colonies. Confirmed *Shigella* and *E. coli* colonies were subsequently sub-cultured in Trypticase Soy Agar (TSA) media for 18 h. Afterward, the colonies were suspended in saline to create an inoculum to obtain an absorbency of around ~ 0.5 McFarland Units. Following this, the samples underwent analysis using VITEK-2 (bioMérieux, France) system. *Shigella* and *E. coli* were identified by the GN-ID card and the AST-N-405 card was used for Antibiotic Susceptibility Test (AST) of the isolates.

The fecal samples were processed for total nucleic acid extraction using the QIAamp Fast DNA Stool Mini Kit and efficiency of nucleic acid extraction and amplification were assessed using MS2 bacteriophage and Phocine herpesvirus (PhHV)^49^. Each sample was tested for *Shigella* ipaH gene by qPCR using SYBR green-based fluorescent dye. PCR was carried out for 40 cycles, with a Ct (cycle threshold) value of 35 designated as the threshold for analysis. A Ct value of ≥ 35 was interpreted as negative^49^.

### Statistical analysis

#### Clinical data

All statistical analyses for clinical data were done in STATA 15 IC (College Station, Texas). Statistical analyses comprised descriptive methods, incorporating numbers and percentages.

The clinical laboratory test results were summarized with median and IQR as data set was not normally distributed and no hypothesis testing was undertaken for these measures due to small number of observations.

#### PK data analysis

Noncompartmental analysis (NCA) was performed on tebipenem PK data using Phoenix® (version 8.3.5) to estimate maximum concentration (C_max_), time of C_max_ (T_max_), and the area under the concentration–time curve (AUC). Tebipenem plasma concentration versus time plot was produced with GraphPad Prism (version 10.0.2). BQL values comprised descriptive methods, incorporating the first measured concentration above the LLOQ were set to 0 for both plotting and PK analysis. All other BQL values were imputed as LLOQ/2 (15 ng/mL) for plotting and excluded from PK analyses. For NCA, missing PK sample concentrations were not imputed.

## Supplementary Information

Supplementary Figure 1.

## Data Availability

Data related to this manuscript are available upon request and for researchers who meet the criteria for access to confidential data may contact with Ms. Shiblee Sayeed (shiblee_s@icddrb.org) to the Research Administration of icddr,b (http://www.icddrb.org/).
